# Influence of the Ovine Genital Tract Microbiota on the Species Artificial Insemination Outcome. A Pilot Study in Commercial Sheep Farms

**DOI:** 10.3390/ht9030016

**Published:** 2020-07-06

**Authors:** Malena Serrano, Eric Climent, Fernando Freire, Juan F. Martínez-Blanch, Carmen González, Luis Reyes, M. Carmen Solaz-Fuster, Jorge H. Calvo, M. Ángeles Jiménez, Francisco M. Codoñer

**Affiliations:** 1 Department of Animal Genetic Improvement, National Institute of Agrarian Technology and Food—INIA, Ctra. de la Coruña, 28040 Madrid, Spain; gverdejo@inia.es (C.G.); angeles.jimenez@inia.es (M.Á.J.); 2 ADM Lifesequencing, University of Valencia Science Park, Carrer del Catedrático A. Escardino Benlloch 9, 46980 Paterna, Spain; Eric.Climent@adm.com (E.C.); Juan.MartinezBlanch@adm.com (J.F.M.-B.); mcarmen.solaz@adm.com (M.C.S.-F.); 3 OVIGEN, Granja Florencia *S*/*N*, Ctra. Villalazán-Peleagonzalo, 49800 Zamora, Spain; gerencia@assafe.es (F.F.); dmalra@hotmail.com (L.R.); 4 Animal Production Technology Unit—CITA, 59059 Zaragoza, Spain; Aragonese Agency Foundation for Research and Development—ARAID, 50004 Zaragoza, Spain; jhcalvo@cita-aragon.es

**Keywords:** microbiota, sheep, reproductive tracts, artificial insemination (AI)

## Abstract

To date, there is a lack of research into the vaginal and sperm microbiome and its bearing on artificial insemination (AI) success in the ovine species. Using hypervariable regions V3–V4 of the 16S rRNA, we describe, for the first time, the combined effect of the ovine microbiome of both females (50 ewes belonging to five herds) and males (five AI rams from an AI center) on AI outcome. Differences in microbiota abundance between pregnant and non-pregnant ewes and between ewes carrying progesterone-releasing intravaginal devices (PRID) with or without antibiotic were tested at different taxonomic levels. The antibiotic treatment applied with the PRID only altered *Streptobacillus* genus abundance, which was significantly lower in ewes carrying PRID with antibiotic. *Mageebacillus*, *Histophilus, Actinobacilllus* and *Sneathia* genera were significantly less abundant in pregnant ewes. In addition, these genera were more abundant in two farms with higher AI failure. Species of these genera such as *Actinobacillus seminis* and *Histophilus somni* have been associated with reproductive disorders in the ovine species. These genera were not present in the sperm samples of AI rams, but were found in the foreskin samples of rams belonging to herd 2 (with high AI failure rate) indicating that their presence in ewes’ vagina could be due to prior transmission by natural mating with rams reared in the herd.

## 1. Introduction

Artificial insemination (AI) is a reproductive technique playing an important role in genetic breeding programs of milk ruminants as it: (i) enables progeny tests to predict rams genetic breeding values (EBVs) according to their daughters’ performances; (ii) contributes to connect herds, which is necessary to establish a common genetic basis to compare EBVs among animals of the whole population and (iii) enables dissemination of the genetic improvement achieved by the genetic program to the whole population, using genetically elite rams. However, AI success is variable among livestock species, with sheep being one of the species with the lowest AI pregnancy rates among ruminants, ranging from 30% to 70%, depending on breeds, season and production systems [1,2,3]. Low AI efficiency has a negative economic impact on dairy sheep breeding programs for two main reasons; firstly, it extends the generational interval, thus delaying genetic enhancement and, secondly, it increases the number of rams to be tested to ensure sufficient progeny.

Many known factors, such as ewes’ cervix conformation, the need to use fresh semen to perform cervical inseminations and the lack of knowledge regarding the exact stage of the ewe’s ovulatory cycle at AI, all contribute to variable extents to lowering AI success rates in this species. Furthermore, the knowledge of other factors affecting AI remains scarce, such as the influence exerted by the genital tract microbiome.

Currently, information regarding microbiome composition can be obtained in two ways: (i) culture-based plus Sanger sequencing of individual isolates and (ii) massive genome sequencing-based technology. Most early works dealing with microbiomes description comes from culture-based approaches, where isolated microorganisms were 16S rRNA analyzed to characterize their diversity in each environment. However, only a 1% of microorganisms can be identified by culture-based techniques, resulting in an underestimation of ecosystem diversity and the inability to identify potentially important organisms. Nowadays, massive genome sequencing technologies can reveal bacterial composition based on the same 16S rRNA gene sequencing, targeting certain hypervariable regions within the gene. These serve as a molecular fingerprint at the genus and species level [4,5] and are particularly interesting for non-culturable species and those present in low abundance.

The human genital tract microbiome has been intensively studied regarding sexual diseases and fertility [6,7,8,9]. *Lactobacillus* species (*Lactobacillus crispatus*, *Lactobacillus iners*, *Lactobacillus gasseri* and *Lactobacillus jensenii*) are the predominant genera in the vaginal tract of healthy women (VMB) promoting a suitable environment in the first stages of embryo development [10]. This dominance of Lactobacilli seems to be typical of humans where the relative abundance of lactobacilli is >70%, while in other mammals they rarely comprise more than 1% of the vaginal microbiota [11]. It is known that lactobacilli control the development and growth of other bacteria by producing lactic acid [12,13], which decreases pH values (3.0–4.5). Vaginal epithelial cells tolerate Lactobacilli, which inhibit induction of pro-inflammatory cytokines [13]. Metagenomic tools were used to characterize the relationship between the male seminal microbiome and the classic semen analysis results in humans. Hou and colleagues [14] analyzed samples obtained from infertile patients and semen donors showing that only *Anaerococcus* genera were related to abnormal sperm parameters. Weng and coworkers [7] showed that the most abundant genera in all sperm samples were *Lactobacillus, Pseudomonas, Prevotella* and *Gardnerella*, finding higher proportions of *Lactobacillus* and *Gardnerella* in normal samples and *Prevotella* in low-quality samples.

Vaginal microbiota samples of ewes and cows with different pregnancy status and age were analyzed by Swartz and coworkers [15]. On sequencing the V3 and V4 regions of the 16S rRNA gene, these authors found a predominance of *Aggregatibacter* spp., *Streptobacillus* spp., *Cronobacter* spp., *Phocoenobacter* spp., and *Psychrilyobacter* spp. genera in ewes, while *Aggregatibacter* spp., *Streptobacillus* spp., *Phocoenobacter* spp., *Sediminicola* spp. and *Sporobacter* spp. were the major genera in cows. No significant differences were found in vaginal microbiota composition of cows between fertilization or embryo-transplantation methods. Microbiota from unmated ewes and cows did not differ from that of mated or pregnant animals, nor were there differences between samples from unmated ewes and recently mated ones. The low abundance of *Lactobacillus* spp. detected [15] is consistent with the neutral pH observed in both the bovine and ovine vagina (7.3 and 6.7, respectively). Cow microbiota showed greater diversity compared to ewe’s, with Bacteroidetes, Fusobacteria, and Proteobacteria being the dominant phyla. Low abundance of Archaea and lactobacilli were found in both species [15]. Regarding the characterization of the microbiota present in sperm of Holstein bulls, González-Marin and colleagues [16] analyzed the bacterial 16S rRNA gene in thawed straws of conventional semen from six Holstein bulls. They found that high bacterial abundance in bull sperm increases spermatozoid DNA damage by about 20-fold in the first 48 h of incubation. After analyzing the sequences of 60 bacterial colonies, species from six different phyla were detected: Bacteroidetes, Firmicutes, Proteobacteria, Cyanobacteria, Fusobacteria and Actinobacteria. In rams, former studies have focused on characterizing the microbiota linked to certain diseases and infertility. *Actinobacillus seminis* was isolated from sheep [17,18] and reported as the cause of epididymitis in rams and abortion in ewes.

The aim of this pilot study is to characterize the vaginal and sperm microbiota of ewes and rams by sequencing the hypervariable regions V3 and V4 of the 16S rRNA gene [19], in order to disentangle its role in AI success, measured as the pregnancy rate observed by ultrasound 42 days after AI. Also, the use of antibiotics was explored to evaluate its effects on the vaginal microbiota and the AI pregnancy rate.

## 2. Materials and Methods

### 2.1. Animal Samples and Experimental Design

Fifty ewes from five different herds (ten ewes per herd) aged from 1.5 to 9 years were used in this experiment. All ewes had lambed at least once. Ewes’ estrous cycles were synchronized with fluorgestone (20 mg) released by intravaginal devices (PRID Chronogest. MSD Animal Health, Kenilworth, NJ, USA) for 15 days, which is the standard AI procedure in genetic breeding programs of commercial dairy sheep breeds. In each herd, five ewes were synchronized with PRID administered with antibiotic (Framycetin in powder 0.6 gr/PRID) (PAB) and five with PRID without antibiotic (PNAB). When PRID were removed ewes were injected with a dose of 300 to 500 mg of PMSG (Pregnant mare’s serum gonadotropin) depending on body weight. AI was conducted 53–55 h after PRID removal. Fresh semen from five AI rams from the artificial insemination center was used to perform cervical inseminations of all 50 ewes. Rams used for AI were aged from 47 to 84 months. Sperm doses were prepared with fresh semen at a concentration of 400 million of spermatozoids/mL using as diluent INRA96^®^ (IMV Technologies, L’Aigle, France) plus 50 mg of streptomycin and 50,000 IU/mL of diluent of penicillin and packed in 25 mL straws. We also analyzed the microbiota of 25 rams from one herd with low AI success, by collecting foreskin mucosa samples and pH measurements. Supplementary Table S1 shows data of the animal samples used in this work.

All ewes (*n* = 50) from five herds located near the AI center were inseminated on the 8 March 2017. Ten ewes from each herd were inseminated with sperm doses from five AI males. Two sperm doses from each male were used to make paired inseminations of one ewe with PAB and one ewe with PNAB in each herd to avoid biases due to the ram effect (Supplementary Figure S1). Inseminations were performed within 5 h of sperm straw preparation, maintaining semen straws at 15 °C. Pregnancy of ewes was detected by ultrasound 42 days after AI.

We also analyzed the foreskin microbiota of 25 natural mating rams from one of the herds with low AI success. All the ewes and rams included in the experiment belong to the same ovine dairy breed.

The current study was carried out under a Project License from the INIA Scientific Ethic Committee. Animal manipulations were performed according to the Spanish Policy for Animal Protection RD 53/2013, which meets the European Union Directive 86/609 about the protection of animals used in experimentation. We hereby confirm that the INIA Scientific Ethic Committee (IACUC) has approved this study.

### 2.2. Microbiome Samples and Microbial DNA Extraction

Before AI, two samples of vaginal exudate from each ewe were collected with sterile swabs (BBL™ CultureSwab™ Liquid Amies (Becton Dickinson, MD, USA), Single Swab for throat, vaginal, skin, and wound specimens BD). Swabs for microbiome DNA extraction were immediately frozen in carbonic snow and subsequently stored at −20 °C until DNA extraction. Rectal temperature was measure in all ewes. From each AI ram, five straws with processed sperm from the same ejaculate used in the AIs were utilized to extract DNA for microbiota analysis. In addition, sterile swabs were to collect samples from the foreskin exudate of 25 natural mating rams of one of the herds with low AI pregnancy rate.

DNA was isolated using the DNeasy Blood and Tissue kit (Qiagen, Hilden, Germany). Prior to DNA isolation, the samples were processed with enzymatic and mechanical treatments in order to ensure the bacterial DNA content outcome. The enzymatic treatment started with 200 µL from the swab mixed with Triton X100 at 1,2% and 200 mg of Silica bead (Biospec, Bartlesville, OK, USA) and a combination of lysozyme (Sigma, Kanagawa, Japan) 74%, Lysostaphin (Sigma, Kanagawa, Japan) 9% and Mutanolysin 10 Ku (Sigma) 17%, this mixture was first incubated for 30 min at 37 °C and 850 rpm. After this enzymatic treatment, the sample was incorporated to the Tissue Lyser (Qiagen, Hilden, Germany) for 5 min at 25 Hz. The DNA isolated following the abovementioned protocol was cleaned and concentrated with the QiAmp Micro kit (Qiagen, Hilden, Germany) to obtain adequate values of quality and concentration.

### 2.3. 16S rRNA Microbiome Sequencing and Bioinformatics

Libraries were prepared following Illumina protocol for 16S rRNA capture and amplification based on dual PCR as implemented by Klindworth and co-workers [19] following Illumina recommendations. Sequencing was performed in a MiSeq Illumina platform yielding 300 nucleotides pair-end reads.

The first step of the bio computation process was the quality control of the sequences. Sequences R1 and R2 were merged in a single read using PEAR program V.0.9.1 (http://www.exelixis-lab.org/web/software/pear) with default options except the overlapping parameter for the sequences of extremes which was fixed at 70 nt. Sequence extremes with low quality (<Q20) and sequences with quality below Q20 were removed. CUTADAPT v1.8.1 software (http://cutadapt.readthedocs.org) was used to remove adapters and select sequences measuring more than 200 nt in length.

Subsequently, the software UCHIME [20] was used to remove chimeric sequences obtained in the amplification process (Supplementary Table S2). DNA sequences from the host (ewe and ram) were also found given the origin of the biological samples (vaginal exudates and sperm). These sequences had to be removed because they would appear as “NoHit” since they could not be assigned to any bacterial taxon. Therefore, previous to the BLAST step comparison with the microorganism database (NCBI RefSeq 16S Microbial database), sequences were compared against the sheep genome (Oar v4.0 reference assembly, annotation release 102) using BLAST local alignment strategy, and those having high similitude were removed from the analysis. Supplementary Table S2 shows the final number of sequences, which were used for the annotation process.

To reduce annotation process complexity, sequences sharing 97% of similitude were grouped in a single sequence with the “cd-hit” software (http://weizhongli-lab.org/jcvi). Finally, sequences were compared with the NCBI RefSeq 16S Microbial database with the BLAST local alignment strategy to associate sequences with each taxonomic group. A rarefaction curve was developed for each sample examined to compare the number of sequences analyzed and the number of taxa detected (Supplementary Figure S2). Thus, the higher the number of sequences, the higher the number of microorganisms detected, until reaching a saturation level where, theoretically, all the organisms have been detected (plateau). If the curve is in an exponential phase, more sequences are needed to be able to detect al the variability contained in the sample.

Microbial 16S rRNA gene composition and diversity were compared among samples at the phyla, family, genera, and species levels. Diversity measure and total richness were determined by calculating the Shannon and the Chao diversity indexes, respectively [21,22].

### 2.4. Statistical Analyses of Phenotypic Data

To study differences in microorganism composition (at the phylum, family, genera and species level) of vaginal samples according to rectal temperatures (in ranges of 0.5 °C), PRID treatment (non-antibiotic NAB, antibiotic AB), ewes’ age, herd management (5 herds) and pregnancy status (non-pregnant, pregnant), we analyzed differential abundance using the DESeq2 R package [23,24]. As herd management practices greatly affect AI outcome, this effect was included as a factor in all comparisons performed with DESeq2. In addition, differences in sperm microbiome composition of AI (AI center) and natural mating (herd 2) rams were evaluated. We conducted pairwise comparisons and calculated the log2FC of the normalized average counts quotient. To test significance of the different effects a T test was performed over the log2FC and its standard error ratio (stat). Statistical *p*_values were adjusted to account for multiple tests [25].

## 3. Results

### 3.1. AI Results

Table 1 shows percentages of AI success rates for the 50 inseminated ewes, the five AI rams and the five herds. Twenty ewes showed positive ultrasound while 30 were negative. Ewes’ rectal temperature ranged between 38.1 and 40.1 °C and averaged 39.2 ± 0.42 °C.

### 3.2. Sequencing Overview

Samples taken from all fifty ewes and semen doses used in the AI trial were processed to characterize ovine vaginal and sperm microbiota. Both vaginal and sperm samples were used to generate a deep V3–V4 16S rRNA gene profile. Consistently, rarefaction curves tended towards an asymptote (Supplementary Figure S2) showing that there were enough sequences to detect all phyla, families, and genera. Chimeras, host sequences and bacterial sequences for each sample are summarized in Supplementary Table S2.

### 3.3. α Diversity

**Figure 1:**
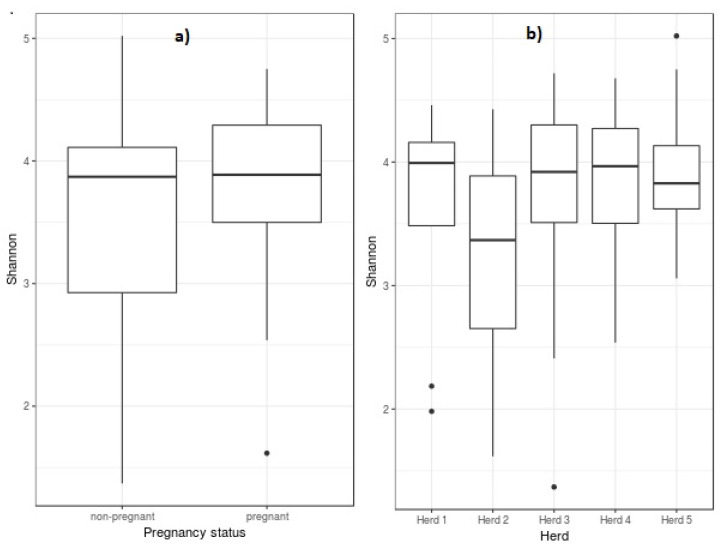
Box plot of Shannon diversity of ewes’ vaginal microbiota at the genus level as compared to pregnancy status (**a**) and herds (**b**). Box lower part represents the lower quartile, box upper part represents the upper quartile, and the bar inside is the median. The whiskers are the minimum and maximum data values, and outliers are shown as small circles.

The Shannon diversity index values indicated moderate diversity of communities at the genus level for vagina (3.88 [3.40–4.21]) and sperm (3.33 [3.22–3.40]), showing the median and interquartile range in brackets. Figure 1 shows the Shannon diversity index at the genus level for pregnancy status (a) and for herds (b). Across herds, Shannon diversity index ranged from 3.37 [2.65–3.89] to 3.99 [3.49–4.16], and differences among them were not significant. Also, non-significant differences were detected between the Shannon diversity index for pregnant (3.89 [3.5–4.29]) and non-pregnant (3.87 [2.93–4.11]) ewes.

A search for the closest species based on sequence similarity obtained in the sequencing step and the bacterial species in the database, enabled us to associate reads to bacterial species and measure a Chao 1 richness index for each sample (Supplementary Figure S3). The Chao richness estimates for herds ranged from 442 ± 110 (herd 1) to 705 ± 522 (herd 5), being the average value 554 ± 2808. Pairwise comparisons between Chao mean values for herds were not significant; however, significant differences were found for variances for pairs herd1-herd3 (*p* = 0.04), herd1-herd5 (*p* = 0.00008), herd2-herd5 (*p* = 0.003), herd3-herd4 (*p* = 0.02), herd3-herd5 (*p* = 0.02) and herd4-herd5 (*p* = 0.00003). Variation coefficients for herds 1, 2, 3, 4 and 5 were 25%, 34%, 36%, 20% and 74%, respectively. No significant differences were found for Chao richness means between non-pregnant (585 ± 340) and pregnant ewes (509 ± 154), but variance differences were highly significant (F = 4.87; *p* = 0.0006). Variation coefficients were 58% and 30% in non-pregnant and pregnant ewes, respectively (Supplementary Figure S3).

### 3.4. General Taxonomic Compositional Traits

Our sequencing approach detected bacterial microbiota but did not reveal species from the archaeal domain. Supplementary Figures S4a and S5 show the most abundant phyla of sperm and vaginal microbiota, respectively. In vaginal (V) and sperm (S) samples the three main phyla were Firmicutes (V = 25% and S = 33%), Actinobacteria (V = 23% and S = 22%) and Proteobacteria (V = 16% and S = 34%). Fusobacteria and Bacteroidetes abundance were V = 6% but only S = 0.2% to 0.4%. Deinococcus–Thermus abundance was V = 0.02% and S = 5%. The most abundant phyla were present in both sexes.

At the family level, vagina and sperm had 15 of the most abundant families in common. Supplementary Figures S4b and S6 show the most abundant families found in sperm and vaginal samples, respectively. The Corynebacteriaceae family showed the highest relative abundance in both sexes (10% to 11%). Pseudomonadaceae was the second most common family found in sperm (S = 10%) while relatively low abundance in vagina (V = 1%). Streptococcaceae was eight times more abundant in rams than in ewes, while Staphylococcaceae showed similar abundance in both sexes (4%).

**Figure 2:**
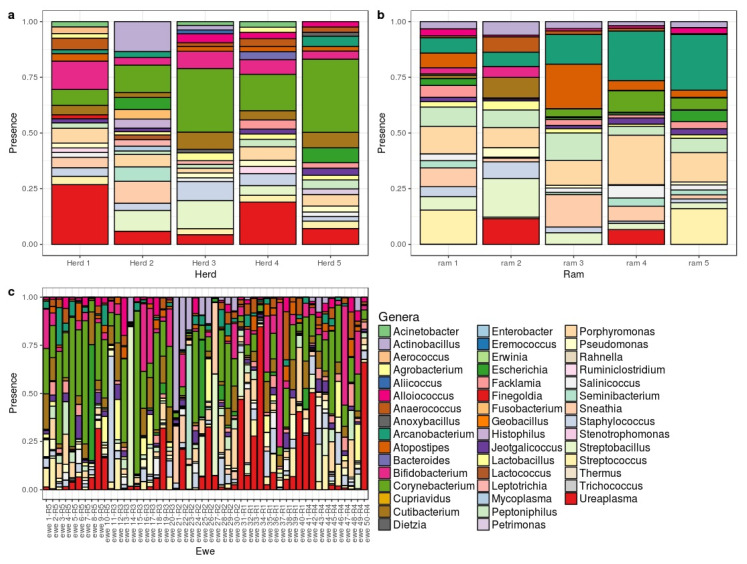
Bar chart showing the distribution of abundant genera for vaginal samples by herd (**a**); sperm sample by ram (**b**) and vaginal microbiome by ewe (**c**).

All genera and species detected in V and S samples are shown in Supplementary Tables S3 and S4, respectively. Figure 2b,c show the most abundant genera found in sperm and vaginal samples, respectively. At the genus level, *Corynebacterium* showed the highest relative abundance in both sample types, representing V = 10% and S = 11% of total counts. *Ureaplasma* was the second most common genus in vagina (6%) and was also present in sperm samples (S = 3%). By contrast, Pseudomonas abundance was S = 10% and just V = 1%. There were many sex-specific genera ranging from 1% to 5.5% of abundance.

Figure 2a show the most abundant genera in ovine vaginal microbiota in each herd. *Ureaplasma* and *Corynebacterium* were present in ewes from all herds at moderately high percentages (2.3 to 13% and 3.4 to 17%, respectively). Results for herd 2 revealed high percentages of *Actinobacillus* (9%) and *Sneathia* (6.6%); these genera were underrepresented in the other herds. In herd 3 *Actinobacillus* was found at a low percentage, 1.1%, while *Sneathia* was recorded at 2.2% in herd 1.

### 3.5. Pairwise Comparisons Between Levels of Effects

Only pairwise comparisons between PRID treatments, pregnancy status and herds showed significant values (*p*_adj) at the phylum, family, genus, and species levels (Supplementary Table S5). Since the PCR product for bacteria yields only 400 nt which did not allow precise species identification, profiles can only be studied at the genus level. Species levels previously reported were shown as the closest found in the database, but this cannot be fully affirmed since a really small portion of the genome is under study. However, putative species enable us to speculate about the impact of their presence in the sample and the observed differences.

#### 3.5.1. PRIDs Treatment with Antibiotic

Table 2 shows significant pairwise comparisons between PRID treatments and microorganism abundance at genus level. Comparison of microorganism abundance between PRID treatments showed that the *Stretobacillus* genus was 21 times more abundant in ewes carrying PRIDs without antibiotic, representing almost 70% of the microorganisms detected in one ewe. No significance for PRID treatment was found at the species level.

#### 3.5.2. Pregnancy Status

**Figure 3:**
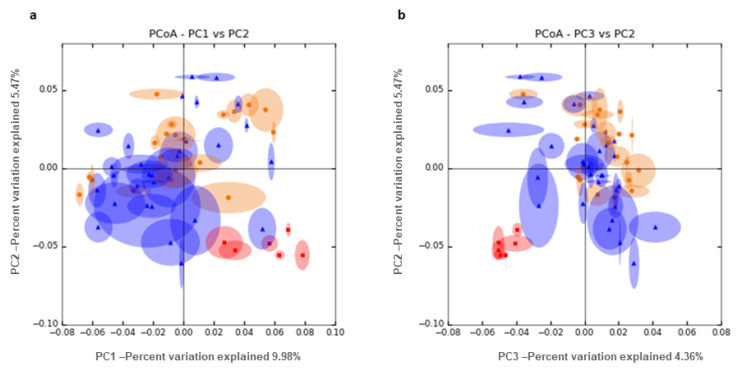
PCoA unweighted UniFrac distance analysis between semen (red) and vaginal samples, taking into account pregnancy outcome, blue (pregnant) and orange (non-pregnant). We show the combination of PC1 and 2 (**a**) and PC2 and 3 (**b**).

We performed a PCoA of unweighted UniFrac distance analysis (Figure 3) comparing semen and vaginal samples (including the differentiation between pregnant and non-pregnant ewes). We can see clear differences between semen, pregnant and non-pregnant samples based on the comparison between PC2 and PC3. Table 2 shows significant pairwise comparisons between pregnancy status and microorganism abundance in ovine vaginal samples at the genus level. Contrasts between pregnancy vs. non-pregnancy status showed four significantly more abundant genera in non-pregnant ewes with a fold change greater than 3. *Mageebacillus*, *Histophilus*, *Actinobacilllus* and *Sneathia* were 70, 25, 22 and 13 times more abundant in non-pregnant than in pregnant ewes. By contrast, the genus *Fluviicola* was 10 times overrepresented in pregnant ewes. The closest species assessed for these genera were *Mageeibacillus indolicus* (log2FC = −4.72; *p*_adj = 0.09) *Histophilus somni* (log2FC = −5.36; *p*_adj = 0.05), *Actinobacillus seminis* (log2FC = −4.80; *p*_adj = 0.03) and *Sneathia sanguinegens* (log2FC = −4.35; *p*_adj = 0.05), which were 41, 28, 26 and 20 times more abundant in non-pregnant than in pregnant ewes.

#### 3.5.3. Herd Comparison

The most significant pairwise comparisons between herds involved herd 2 versus herds 1, 3, 4 and 5. Table 3 shows significant pairwise comparisons for microorganism abundance between herds at the genus level. Genera overrepresented in herd 2 were *Histophilus* (200–2455 times more abundant), *Sneathia* (160–782 times more abundant), *Mageeibacillus* (90–323 times more abundant) and *Actinobacillus* (89–570 times more abundant). In addition, Actinobacillus was 236 times more abundant in herd 3 vs. herd 5. Putative species detected for these genera were *Histophilus somni* (log2FC from 8.5 to 10.5, *p*adj < 0.0001), *Sneathia sanguinegens* (log2FC from 7.1 to 9.5, *p*adj < 0.0001), *Mageeibacillus indolicus* (log2FC from 6.5 to 7.7, *p*adj < 0.01), and *Actinobacillus seminis* (log2FC from 6 to 9, *p*adj < 0.005).

### 3.6. Natural Mating Rams Microbiome in Herd 2

After confirming the absence of the putative bacteria promoting AI failure in the AI rams, we decided to study the foreskin microbiota in a cohort of 25 natural mating rams from herd 2, whose ewes harbored greater abundance of genera related to pregnancy failure, in order to confirm whether those rams harbored these genera. We obtained a mean count of 58,300 sequences for each ram analyzed, the exact number for each sample can be found in Supplementary Table S2. We verified the presence of those bacteria that seem to be responsible for pregnancy failure (Supplementary Figure S7), *Actinobacillus* and *Sneathia* genera, in most of the analyzed rams (60 and 56% of the rams, respectively). Also, we confirmed the presence of *Histophilus* and *Mageebacillus* in some of the natural mating analyzed rams (more information about genera and species detected can be seen in Supplementary Tables S3 and S4). Contrast of microbiota abundance between natural mating rams under 20 months old and those over 20 months revealed that *Actinomyces* (log2FC = −3.47; *p*-adj = 0.03) and *Staphylococcus* (log2FC = −4.26; *p*-adj = 0.001) were significantly more abundant in young males, and *Muribaculum* (log2FC = 9.96; *p*-adj = 8.22 E-06), *Lactobacillus* (log2FC = 6.14; *p*-adj = 0.0001), *Lachnoclostridium* (log2FC = 5.58; *p*-adj = 0.0004) and *Bacteroides* (log2FC = 3.60; *p*-adj = 0.01) in rams older than 20 months (Supplementary Table S5). No differences in the abundance of those genera previously associated with pregnancy failure were found between rams aged >20 months and those aged ≤20 months. In natural mating rams no differences in microbiota composition were found when considering pH as differential factor.

## 4. Discussion

From the very outset of this work, we have been well aware that the use of PRIDs containing fluogestone to synchronize ewes’ estrus and the preparation of rams’ sperm straws, can modify the microbiota of vaginal [9,26] and sperm samples. However, we have performed this study using intravaginal devices and conventional sperm straws because our main aim was to detect the microbiota associated to AI failure, and AIs in commercial dairy ewes are always performed in this way. In Spain, the use of antibiotics in the AI sperm straws is mandatory for livestock species. In addition, many farmers use antibiotic in the PRIDs to avoid infections derived from its use. Another limitation of this work came from working with commercial herds where experimental sample sizes are restricted.

Shannon diversity index of genera found in vaginal samples from ewes (3.62 ± 0.80) was similar to that observed in the Rambouillet breed (2.87 ± 1.16) [15] and did not show any association with either the ewe’s pregnancy status or with herds’ AI success rate. However, Chao richness index showed significant lower variance in those herds with higher AI success rates (herd 1 and herd 4). Thus, higher dispersion in the abundance of genera seems to have relationship with reproductive failure in AI.

In the work by Swartz and coworkers [15], the predominant genera found in ewes’ vaginal tract were *Aggregatibacter*, *Streptobacillus*, *Cronobacter*, *Phocoenobacter* and *Psychrilyobacter*. None of these genera except *Streptobacillus* were found in our study. Our results indicate that *Streptobacillus* was the only genus affected by the antibiotic (Framycetin) used in the PRIDS, since it was 21 times (*p*_adj = 0.0002) overrepresented in ewes carrying PRIDS without antibiotic. Moreover, we detected a non-significant decrease in *Proteobacteria* and *Clostridia* species, which would affect microbiome stability. One species of this genus, *Streptobacillus notomytis*, was 19 times more abundant (*p*_adj = 0.05) in non-pregnant than in pregnant ewes. These results show the antibiotic Framycetin is quite effective in eliminating one bacterial genus associated with a certain degree of reproductive failure. The antibiotic used with the PRIDs did not affect the growth of the remaining microorganisms detected in ewes’ vaginal samples.

*Mageeibacillus*, *Histophilus*, *Actinobacillus*, and *Sneathia*, are the main significantly overrepresented genera (>3 log2FC) in non-pregnant versus pregnant ewes and in two of the herds with lower AI success (herds 2 and 3). Our results did not find any relationship between the abundance of *Actinobacillus* and *Histophilus* and ewes age previously described by Walker and colleagues (Walker et al. 1986).

*Mageeibacillus*, a genus belonging to the order *Clostridiales* and the family *Ruminococcaceae*. *Mageeibacillus indolicus,* has been detected in the vaginal tract of women with bacterial vaginitis [27]. In our work this genus was nearly 70 times more abundant in non-pregnant than in pregnant ewes, although its base mean was very low. To date, there is a lack of information about the presence and effect of this genus on livestock.

*Histophilus* and *Actinobacillus* belong to the Pasteurellaceae family (phylum Proteobacteria) and to the HAP group (*Haemophilus-Actinobacillus-Pasteurella*) which includes a great number of human and livestock pathogens resistant to most cellular defense mechanisms [28]. *Actinobacillus seminis* has been related with abnormal semen, epididymitis, and infertility [18,29,30] in rams, abortion in ewes [17] and vaginitis [31] and Purulent Vaginal Discharge in cows [32]. Epididymitis results in substantial economic losses worldwide due to reproductive failure and related culling. The most common causative agents of these infections are *Brucella ovis*, *Actinobacillus seminis*, and *Histophilus somni* [33]. Epididymitis is a contagious disease of venereal or homosexual transmission and in the case of *Actinobacillus seminis*, it is transmitted from ewe to lamb [34]. However, in the sperm straws of the five AI rams used in this work, neither *Actinobacillus* nor *Histophilus* were detected. Furthermore, we did not find any relationship between AI rams’ microbiota and AI success/failure.

The abundance of these genera in the vaginal tract of non-pregnant ewes, and more strikingly in ewes from herd 2 with low AI success, could be due to the transmission of these microorganisms by the natural mating with rams reared in the herd. This hypothesis is plausible since all ewes have at least one natural lambing before AI is performed. In order to corroborate the relationship between AI failure and previous natural mating with rams in the herd, we analyzed the foreskin microbiota of 25 rams from one of the herds with low fertility success (herd2, 20% AI success) and with high presence of the abovementioned genera in its ewes. In the natural mating rams from herd 2, we found the same bacterial genera that have been associated with AI failure in the ewes’ vaginal samples.

Bacteria of the genus *Sneathia* are potential pathogens in the female reproductive tract. Species of *Sneathia* were initially grouped with *Leptotrichia*, but in a study based on phylogenetic and phenotypic analyses, the organism previously named *Leptotrichia sanguinegens* was reassigned to the genus *Sneathia* and named *Sneathia sanguinegens* [35]. *Sneathia* is part of the normal microbiota of the genitourinary tracts of both men and women, but they have been also associated with some clinical diseases such as bacterial vaginosis [36], spontaneous abortion and other infections [37]. However, to date, there is no information about the presence of this genus in the ovine reproductive tract or about its relationship with reproductive efficiency in this species.

In our work, the genus *Ureaplasma* did not present a significant association with the pregnancy success rate. The species *Ureaplasma diversum* has been associated with bovine reproductive illnesses and in particular with granular vulvo-vaginitis [11]. Also, this species has been identified in AI bovine sperm straws and seems to be related with abnormal spermatic morphology, clumping and venereal transmission to herds [38]. Here we found higher abundance of *Ureaplasma diversum* in herds with high pregnancy rates (13% in herd 1 with 70% pregnancy rate, and 8% in herd 4 with 60% pregnancy rate) than in those with a low AI success rate (from 2% to 4%). Therefore, at least in the ovine species, the *Ureaplasma* genus does not seem to affect AI fertility.

In view of the results reported here, we can state that a proportion of the differences observed in the percentage of AI success among herds could be explained by the composition and abundance of the vaginal microbiota in ewes, but also, that management practices in each herd greatly contributed to the variability of the AI pregnancy rate.

Future experimental work will be performed to assess the impact of the transmission of microorganisms from natural mating rams to ewes and to investigate whether this transmission can lead to reversible or permanent changes in the vaginal microbiota of ewes, which would imply a serious constraint for the future AI success. Molecular diagnosis of *Actinobacillus seminis* and *Histophilus somni* could be performed routinely by conventional multiplex PCR [33] in ewes and rams to determine their abundance at the individual, farm and population levels.

The influence of the host genome on the observed differences in the vaginal tract and sperm microbiota composition and its relationship with fertility in the ovine species should also be studied in order to detect key genomic regions and genes, facilitating the genetic selection of animals, and thereby promoting beneficial microbiota for fertility and reproductive success.

Furthermore, probiotics and prebiotics can be used to indirectly manipulate microbiome composition [26,39]. We speculate that the incorporation of probiotics such as *Bifidobacterium* and *Lactobacillus* in the ovine vagina would inhibit the growth of potentially pathogenic bacteria such as *Histophylus*, *Actinobacteria*, *Mageeibacillus* and *Sneathia*. In rams’ sperm, *Lactobacillus* could be a potential probiotic for semen quality maintenance and would help counteract the negative effects of noxious bacteria [7].

The constraints of this study mentioned above, make this work a preliminary approach to this topic. Future studies including a high number of samples and a more robust experimental design, will be conducted to confirm the results here addressed.

## 5. Conclusions

In summary, our results show that the microbial composition of the ovine genital tract does influence AI success. We can affirm that microbiota composition of the ewe’s genital tract is primarily responsible for AI success/failure, whereas sperm microbiota of AI rams did not show any relationship with AI outcome. Four genera were found to play a significant role in ovine AI success, i.e., *Mageebacillus*, *Histophilus*, *Actinobacilllus* and *Sneathia*, since their high relative abundance in vaginal samples were clearly associated with AI failure. Most of these genera, which are typical of reproductive diseases affecting rams, were not present in the sperm of AI rams, but they were found in foreskin samples of natural mating rams in the herds under study. Thus, we hypothesize that venereal transmission of these microorganisms takes place during natural mating of ewes with rams reared in herds. The use of antibiotic in the PRIDs seems to be ineffective in eliminating the genera more closely related with AI failure, and could be harmful for the maintenance of a well-balanced vaginal microbiota, especially in those herds with higher counts of putative harmful bacteria.

## Figures and Tables

**Table 1:** Percentage of fertility by artificial insemination for ewes, rams and herds involved in the experiment.

Animals	% AI Success
AI Ram 1	40
AI Ram 2	40
AI Ram 3	40
AI Ram 4	30
AI Ram 5	50
Ewes	40
Herd 1	70
Herd 2	20
Herd 3	20
Herd 4	60
Herd 5	30

**Table 2:** Significant contrasts of pairwise comparisons between PRID treatments (PNAB = PRID without antibiotic/PAB = PRID with antibiotic) and pregnancy status (P = pregnant/NP = non-pregnant) at genus level. stat = log2FC/log2FC_SE_. *p*_adj = adjusted *p*_value for multiple tests.

**PRID**	**Kingdom, Phylum, Class, Order, Family and Genera**	**log2FC**	**log2FCse**	**Stat**	***p*_value**	***p*_adj**
PNAB vs. PAB	Bacteria; Fusobacteria; Fusobacteriia; Fusobacteriales; Leptotrichiaceae; *Streptobacillus*	4.424	0.854	5.183	2.18 × 10^−7^	0.0002
**Pregnancy**	**Kingdom, Phylum, Class, Order, Family and Genera**	**log2FC**	**log2FCse**	**stat**	***p*_value**	***p*_adj**
P vs. NP	Bacteria; Bacteroidetes; Flavobacteriia; Flavobacteriales; Crocinitomicaceae; *Fluviicola*	3.297	1.005	3.282	0.001030	0.02988
P vs. NP	Bacteria; Actinobacteria; Actinobacteria; Streptomycetales; Streptomycetaceae; *Streptomyces*	1.348	0.491	2.745	0.006057	0.09989
P vs. NP	Bacteria; Actinobacteria; Actinobacteria; Corynebacteriales; Corynebacteriaceae; *Corynebacterium*	−1.477	0.400	−3.694	0.000221	0.01318
P vs. NP	Bacteria; Proteobacteria; Gammaproteobacteria; Enterobacterales; Enterobacteriaceae; *Escherichia*	−2.825	0.836	−3.380	0.000725	0.02988
P vs. NP	Bacteria; Firmicutes; Erysipelotrichia; Erysipelotrichales; Erysipelotrichaceae; *Erysipelothrix*	−3.223	1.176	−2.740	0.006138	0.09989
P vs. NP	Bacteria; Bacteroidetes; Bacteroidia; Bacteroidales; Porphyromonadaceae; *Petrimonas*	−3.245	0.988	−3.285	0.001022	0.02988
P vs. NP	Bacteria; Fusobacteria; Fusobacteriia; Fusobacteriales; Leptotrichiaceae; *Sneathia*	−3.757	1.190	−3.158	0.001587	0.03551
P vs. NP	Bacteria; Proteobacteria; Gammaproteobacteria; Pasteurellales; Pasteurellaceae; *Actinobacillus*	−4.492	1.114	−4.033	5.51 × 10^−5^	0.00494
P vs. NP	Bacteria; Proteobacteria; Gammaproteobacteria; Pasteurellales; Pasteurellaceae; *Histophilus*	−4.663	1.436	−3.246	0.001168	0.02988
P vs. NP	Bacteria; Firmicutes; Clostridia; Clostridiales; Ruminococcaceae; *Mageeibacillus*	−6.121	1.340	−4.568	4.92 × 10^−6^	0.00088

**Table 3:** Significant contrasts of pairwise comparisons between herds at genus level. stat = log2FC/log2FC_SE_. *p*_adj = adjusted *p*_value for multiple tests.

Herds	Kingdom, Phylum, Class, Order, Family and Genera	log_2_FC	log_2_FCse	stat	*p*_value	*p*_adj
2 vs 1	Bacteria; Proteobacteria; Gammaproteobacteria; Enterobacterales; Enterobacteriaceae; *Escherichia*	5.340	1.158	4.613	3.96 × 10^−6^	0.000291
2 vs 1	Bacteria; Fusobacteria; Fusobacteriia; Fusobacteriales; Fusobacteriaceae; *Fusobacterium*	6.375	1.516	4.207	2.60 × 10^−5^	0.001187
2 vs 1	Bacteria; Proteobacteria; Gammaproteobacteria; Pasteurellales; Pasteurellaceae; *Actinobacillus*	6.480	1.419	4.567	4.94 × 10^−6^	0.000291
2 vs 1	Bacteria; Firmicutes; Clostridia; Clostridiales; Ruminococcaceae; *Mageeibacillus*	6.487	1.596	4.064	4.82 × 10^−5^	0.001626
2 vs 1	Bacteria; Tenericutes; Mollicutes; Mycoplasmatales; Mycoplasmataceae; *Mycoplasma*	7.233	1.734	4.172	3.01 × 10^−5^	0.001187
2 vs 1	Bacteria; Fusobacteria; Fusobacteriia; Fusobacteriales; Leptotrichiaceae; *Sneathia*	7.326	1.357	5.400	6.65 × 10^−8^	1.57 × 10^−5^
2 vs 1	Bacteria; Proteobacteria; Gammaproteobacteria; Pasteurellales; Pasteurellaceae; *Histophilus*	8.162	1.572	5.191	2.08 × 10^−7^	2.46 × 10^−5^
2 vs 4	Bacteria; Firmicutes; Clostridia; Clostridiales; Ruminococcaceae; *Mageeibacillus*	8.337	1.605	5.195	2.04 × 10^−7^	0.000104
2 vs 4	Bacteria; Proteobacteria; Gammaproteobacteria; Pasteurellales; Pasteurellaceae; *Histophilus*	9.501	1.578	6.020	1.70 × 10^−9^	1.79 × 10^−6^
3 vs 2	Bacteria; Actinobacteria; Actinobacteria; Actinomycetales; Actinomycetaceae; *Actinomyces*	−4.919	1.156	−4.256	2.08 × 10^−5^	0.001369
3 vs 2	Bacteria; Fusobacteria; Fusobacteriia; Fusobacteriales; Leptotrichiaceae; *Streptobacillus*	−5.729	1.231	−4.654	3.26 × 10^−6^	0.000321
3 vs 2	Bacteria; Fusobacteria; Fusobacteriia; Fusobacteriales; Leptotrichiaceae; *Leptotrichia*	−6.061	1.455	−4.167	3.08 × 10^−5^	0.001522
3 vs 2	Bacteria; Fusobacteria; Fusobacteriia; Fusobacteriales; Fusobacteriaceae; *Fusobacterium*	−6.422	1.517	−4.232	2.31 × 10^−5^	0.001369
3 vs 2	Bacteria; Proteobacteria; Gammaproteobacteria; Pasteurellales; Pasteurellaceae; *Histophilus*	−7.644	1.569	−4.872	1.10 × 10^−6^	0.000163
3 vs 2	Bacteria; Fusobacteria; Fusobacteriia; Fusobacteriales; Leptotrichiaceae; *Sneathia*	−8.273	1.363	−6.068	1.29 × 10^−9^	3.84 × 10^−7^
5 vs 2	Bacteria; Firmicutes; Bacilli; Bacillales; Staphylococcaceae; *Staphylococcus*	−2.476	0.615	−4.029	5.60 × 10^−5^	0.001520
5 vs 2	Bacteria; Actinobacteria; Actinobacteria; Actinomycetales; Actinomycetaceae; *Actinomyces*	−4.166	1.150	−3.622	0.00029	0.006352
5 vs 2	Bacteria; Firmicutes; Clostridia; Clostridiales; Lachnospiraceae; *Anaerostipes*	−5.022	1.340	−3.749	0.00017	0.004279
5 vs 2	Bacteria; Tenericutes; Mollicutes; Mycoplasmatales; Mycoplasmataceae; *Mycoplasma*	−5.680	1.723	−3.296	0.00098	0.019338
5 vs 2	Bacteria; Proteobacteria; Gammaproteobacteria; Enterobacterales; Enterobacteriaceae; *Escherichia*	−6.109	1.156	−5.283	1.27 × 10^−7^	4.59 × 10^−6^
5 vs 2	Bacteria; Firmicutes; Clostridia; Clostridiales; Ruminococcaceae; *Mageeibacillus*	−6.941	1.590	−4.366	1.26 × 10^−5^	0.000392
5 vs 2	Bacteria; Fusobacteria; Fusobacteriia; Fusobacteriales; Leptotrichiaceae; *Streptobacillus*	−7.201	1.230	−5.853	4.81 × 10^−9^	2.09 × 10^−7^
5 vs 2	Bacteria; Fusobacteria; Fusobacteriia; Fusobacteriales; Leptotrichiaceae; *Leptotrichia*	−8.550	1.460	−5.857	4.70 × 10^−9^	2.09 × 10^−7^
5 vs 2	Bacteria; Proteobacteria; Gammaproteobacteria; Pasteurellales; Pasteurellaceae; *Actinobacillus*	−9.156	1.421	−6.445	1.15 × 10^−10^	8.34 × 10^−9^
5 vs 2	Bacteria; Fusobacteria; Fusobacteriia; Fusobacteriales; Leptotrichiaceae; *Sneathia*	−9.610	1.362	−7.057	1.70 × 10^−12^	1.84 × 10^−10^
5 vs 2	Bacteria; Proteobacteria; Gammaproteobacteria; Pasteurellales; Pasteurellaceae; *Histophilus*	−11.262	1.588	−7.093	1.31 × 10^−12^	1.84 × 10^−10^
5 vs 3	Bacteria; Proteobacteria; Gammaproteobacteria; Pasteurellales; Pasteurellaceae; *Actinobacillus*	−7.885	1.421	−5.551	2.84 × 10^−8^	2.91 × 10^−5^

## Supplementary Materials

Supplementary materials can be found at https://www.mdpi.com/2571-5135/9/3/16/s1. Table S1 Ewes and ram’s data. Table S2 Number of sequences after quality control steps explained in the material and methods section, number and percentage of host sequences and number and percentage of bacterial sequences for each animal analyzed. Table S3 Differential abundance of microorganisms at the genus level for ewes, AI rams and natural mating rams from herd 2. The genera present in the samples are shown in sheet 1, those with counts above 1% are shown in sheet 2. Table S4 Differential abundance of microorganisms at the species level for ewes, AI rams and natural mating rams from herd 2. The species present in the samples are shown in sheet 1, those with counts above 1% are shown in sheet 2. Table S5 Significant pairwise comparison of microorganism abundance among levels of effects at the phylum, family, genus and species levels. Normalized mean of counts, log2FC and its standard error ratio (log2FCse), log2FC/log2FCse ratio (stat), *p*_values and *p*_values (*p*_adj) adjusted for multiple tests (Benjamini and Hochberg). Figure S1 Experimental design. Figure S2 Rarefaction curves of detected taxa at the phylum, family and genus levels in ewes’ vaginal tract and rams’ sperm. Figure S3 Chao richness estimates of ewe’s vaginal microbiota at the genus level as compared to herds a) and pregnancy status b). Box lower part represents the lower quartile, box upper part represents the upper quartile, and the bar inside is the median. The whiskers are the minimum and maximum data values, and outliers are shown as small circles. Figure S4 Bar chart showing the distribution of the most abundant phyla (above) and family (below) of AI rams’ sperm microbiota. Figure S5 Bar chart showing the distribution of the most abundant phyla of ewes’ vaginal microbiota. Figure S6 Bar chart showing the distribution of the most abundant family of ewes’ vaginal microbiota. Figure S7 Bar chart showing the distribution of the most abundant genera present in the herd 2 natural mating rams’ foreskin and ewes’ vaginal microbiota.
