# Supplementary material for: Influence of the Ovine Genital Tract Microbiota on the Species Artificial Insemination Outcome. A Pilot Study in Commercial Sheep Farms
Source: High Throughput. 2020 Jul 6;9(3):16. doi: 10.3390/ht9030016 (PMC7576495; doi:10.3390/ht9030016)

EXPERIMENTAL DESIGN

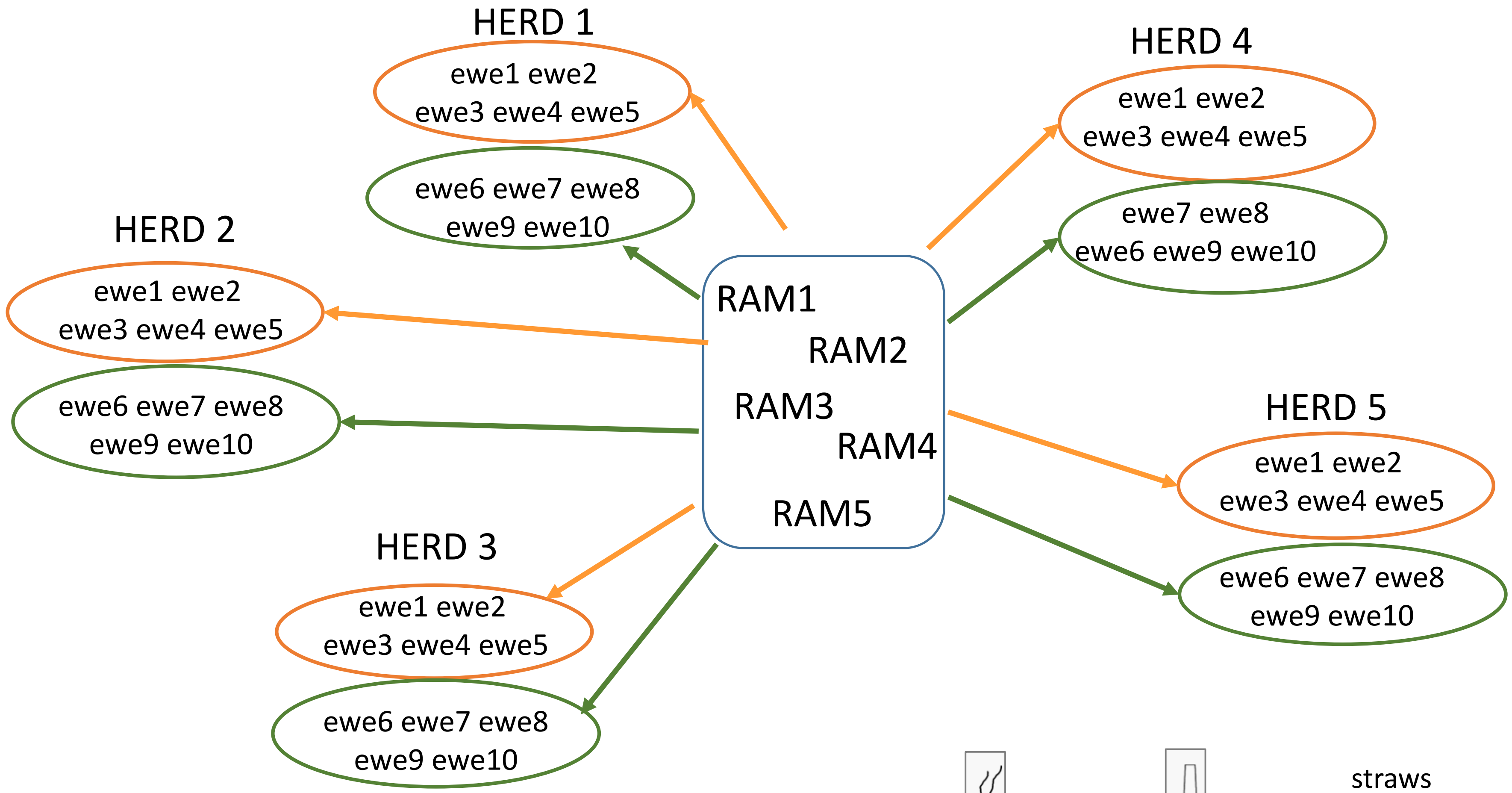

- *5 herds, 10 ewes/herd*
- *Age 1.5 to 6 years*
- *At least one lambing*

5 ewes/herd $\text{PRID}_{\text{ANTIBIOTIC}}$

5 ewes/herd $\text{PRID}_{\text{NON-ANTIBIOTIC}}$

- *5 AI rams*
- *Age 4 to 7 years*
- *Each ram inseminate 10 ewes (2 ewes/herd)*
- *AIs performed by a single veterinarian in the same day*

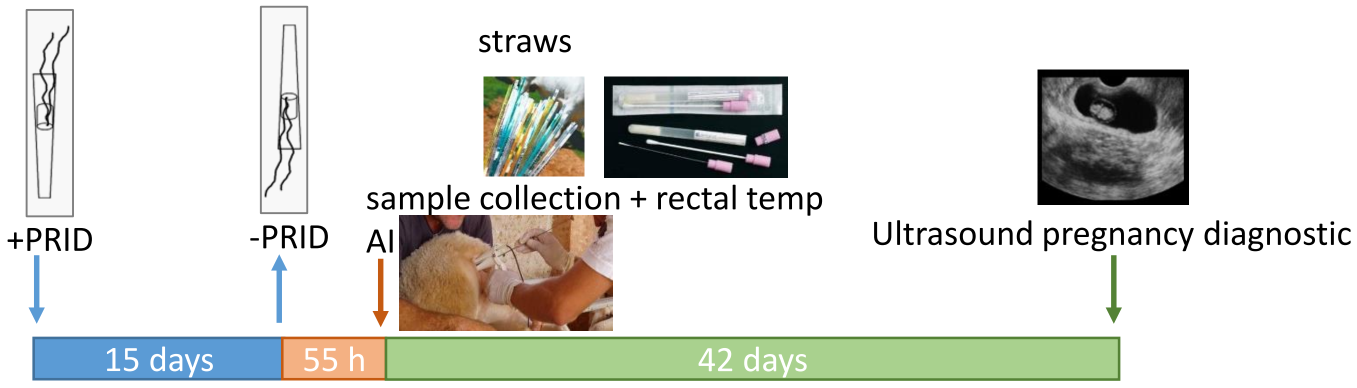

Supplement: Supplementary file 1 [file high-throughput-09-00016-s001.zip › Figure S1.pdf]
