# Supplementary material for: Influence of the Ovine Genital Tract Microbiota on the Species Artificial Insemination Outcome. A Pilot Study in Commercial Sheep Farms
Source: High Throughput. 2020 Jul 6;9(3):16. doi: 10.3390/ht9030016 (PMC7576495; doi:10.3390/ht9030016)
Supplement: Supplementary file 1 [file high-throughput-09-00016-s001.zip › Figure S2.pdf]

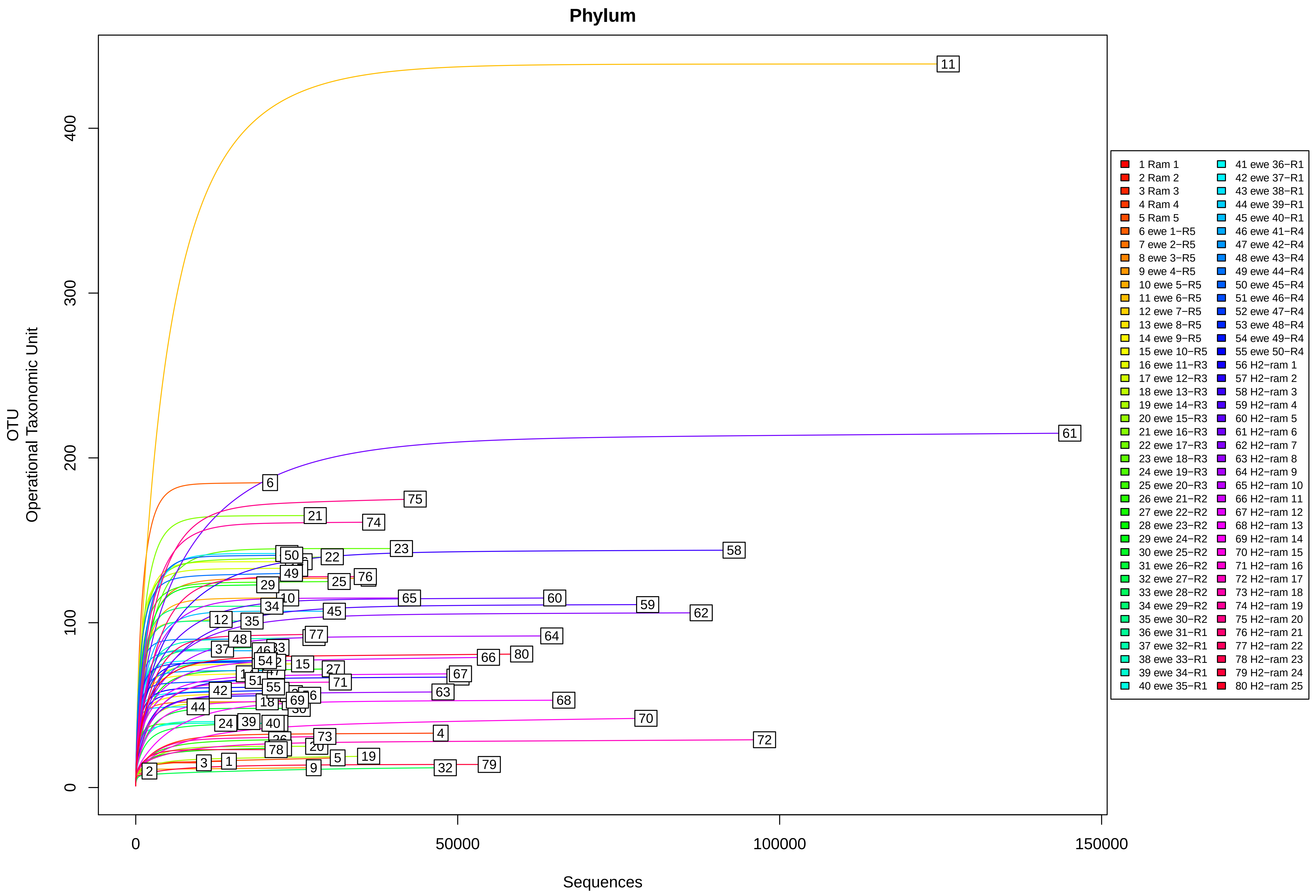
Phylum
OTU
Operational Taxonomic Unit
Sequences
0
100
200
300
400
0
50000
100000
150000
1 Ram 1
2 Ram 2
3 Ram 3
4 Ram 4
5 Ram 5
6 ewe 1−R5
7 ewe 2−R5
8 ewe 3−R5
9 ewe 4−R5
10 ewe 5−R5
11 ewe 6−R5
12 ewe 7−R5
13 ewe 8−R5
14 ewe 9−R5
15 ewe 10−R5
16 ewe 11−R3
17 ewe 12−R3
18 ewe 13−R3
19 ewe 14−R3
20 ewe 15−R3
21 ewe 16−R3
22 ewe 17−R3
23 ewe 18−R3
24 ewe 19−R3
25 ewe 20−R3
26 ewe 21−R2
27 ewe 22−R2
28 ewe 23−R2
29 ewe 24−R2
30 ewe 25−R2
31 ewe 26−R2
32 ewe 27−R2
33 ewe 28−R2
34 ewe 29−R2
35 ewe 30−R2
36 ewe 31−R1
37 ewe 32−R1
38 ewe 33−R1
39 ewe 34−R1
40 ewe 35−R1
41 ewe 36−R1
42 ewe 37−R1
43 ewe 38−R1
44 ewe 39−R1
45 ewe 40−R1
46 ewe 41−R4
47 ewe 42−R4
48 ewe 43−R4
49 ewe 44−R4
50 ewe 45−R4
51 ewe 46−R4
52 ewe 47−R4
53 ewe 48−R4
54 ewe 49−R4
55 ewe 50−R4
56 H2−ram 1
57 H2−ram 2
58 H2−ram 3
59 H2−ram 4
60 H2−ram 5
61 H2−ram 6
62 H2−ram 7
63 H2−ram 8
64 H2−ram 9
65 H2−ram 10
66 H2−ram 11
67 H2−ram 12
68 H2−ram 13
69 H2−ram 14
70 H2−ram 15
71 H2−ram 16
72 H2−ram 17
73 H2−ram 18
74 H2−ram 19
75 H2−ram 20
76 H2−ram 21
77 H2−ram 22
78 H2−ram 23
79 H2−ram 24
80 H2−ram 25

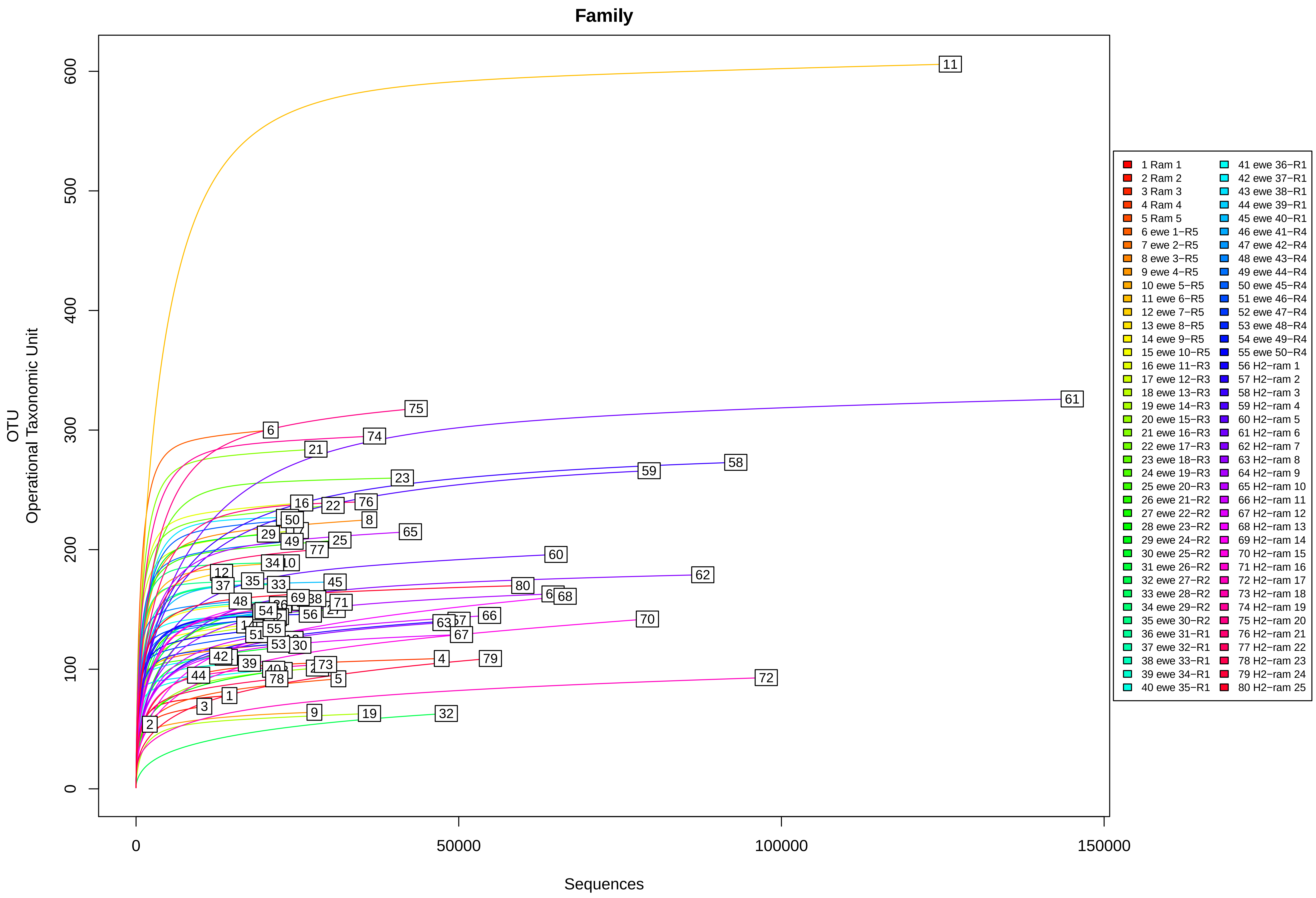
Family
OTU
Operational Taxonomic Unit
Sequences
0
100
200
300
400
500
600
0
50000
100000
150000
1 Ram 1
2 Ram 2
3 Ram 3
4 Ram 4
5 Ram 5
6 ewe 1−R5
7 ewe 2−R5
8 ewe 3−R5
9 ewe 4−R5
10 ewe 5−R5
11 ewe 6−R5
12 ewe 7−R5
13 ewe 8−R5
14 ewe 9−R5
15 ewe 10−R5
16 ewe 11−R3
17 ewe 12−R3
18 ewe 13−R3
19 ewe 14−R3
20 ewe 15−R3
21 ewe 16−R3
22 ewe 17−R3
23 ewe 18−R3
24 ewe 19−R3
25 ewe 20−R3
26 ewe 21−R2
27 ewe 22−R2
28 ewe 23−R2
29 ewe 24−R2
30 ewe 25−R2
31 ewe 26−R2
32 ewe 27−R2
33 ewe 28−R2
34 ewe 29−R2
35 ewe 30−R2
36 ewe 31−R1
37 ewe 32−R1
38 ewe 33−R1
39 ewe 34−R1
40 ewe 35−R1
41 ewe 36−R1
42 ewe 37−R1
43 ewe 38−R1
44 ewe 39−R1
45 ewe 40−R1
46 ewe 41−R4
47 ewe 42−R4
48 ewe 43−R4
49 ewe 44−R4
50 ewe 45−R4
51 ewe 46−R4
52 ewe 47−R4
53 ewe 48−R4
54 ewe 49−R4
55 ewe 50−R4
56 H2−ram 1
57 H2−ram 2
58 H2−ram 3
59 H2−ram 4
60 H2−ram 5
61 H2−ram 6
62 H2−ram 7
63 H2−ram 8
64 H2−ram 9
65 H2−ram 10
66 H2−ram 11
67 H2−ram 12
68 H2−ram 13
69 H2−ram 14
70 H2−ram 15
71 H2−ram 16
72 H2−ram 17
73 H2−ram 18
74 H2−ram 19
75 H2−ram 20
76 H2−ram 21
77 H2−ram 22
78 H2−ram 23
79 H2−ram 24
80 H2−ram 25

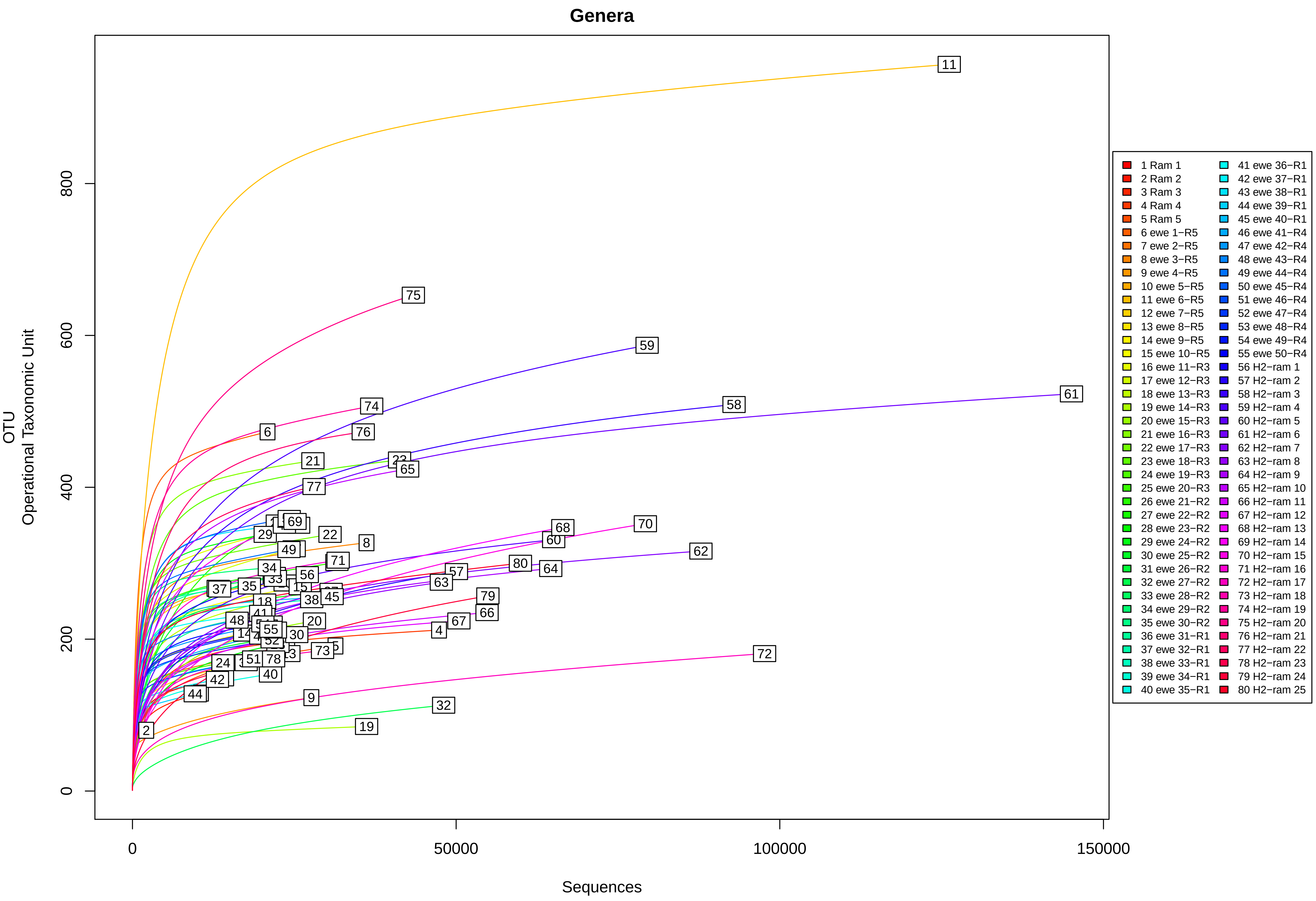
Genera
OTU
Operational Taxonomic Unit
Sequences
0
200
400
600
800
0
50000
100000
150000
1 Ram 1
2 Ram 2
3 Ram 3
4 Ram 4
5 Ram 5
6 ewe 1−R5
7 ewe 2−R5
8 ewe 3−R5
9 ewe 4−R5
10 ewe 5−R5
11 ewe 6−R5
12 ewe 7−R5
13 ewe 8−R5
14 ewe 9−R5
15 ewe 10−R5
16 ewe 11−R3
17 ewe 12−R3
18 ewe 13−R3
19 ewe 14−R3
20 ewe 15−R3
21 ewe 16−R3
22 ewe 17−R3
23 ewe 18−R3
24 ewe 19−R3
25 ewe 20−R3
26 ewe 21−R2
27 ewe 22−R2
28 ewe 23−R2
29 ewe 24−R2
30 ewe 25−R2
31 ewe 26−R2
32 ewe 27−R2
33 ewe 28−R2
34 ewe 29−R2
35 ewe 30−R2
36 ewe 31−R1
37 ewe 32−R1
38 ewe 33−R1
39 ewe 34−R1
40 ewe 35−R1
41 ewe 36−R1
42 ewe 37−R1
43 ewe 38−R1
44 ewe 39−R1
45 ewe 40−R1
46 ewe 41−R4
47 ewe 42−R4
48 ewe 43−R4
49 ewe 44−R4
50 ewe 45−R4
51 ewe 46−R4
52 ewe 47−R4
53 ewe 48−R4
54 ewe 49−R4
55 ewe 50−R4
56 H2−ram 1
57 H2−ram 2
58 H2−ram 3
59 H2−ram 4
60 H2−ram 5
61 H2−ram 6
62 H2−ram 7
63 H2−ram 8
64 H2−ram 9
65 H2−ram 10
66 H2−ram 11
67 H2−ram 12
68 H2−ram 13
69 H2−ram 14
70 H2−ram 15
71 H2−ram 16
72 H2−ram 17
73 H2−ram 18
74 H2−ram 19
75 H2−ram 20
76 H2−ram 21
77 H2−ram 22
78 H2−ram 23
79 H2−ram 24
80 H2−ram 25
