# Supplementary material for: Influence of the Ovine Genital Tract Microbiota on the Species Artificial Insemination Outcome. A Pilot Study in Commercial Sheep Farms
Source: High Throughput. 2020 Jul 6;9(3):16. doi: 10.3390/ht9030016 (PMC7576495; doi:10.3390/ht9030016)

a) Chao Richness Estimation for herd

herd	*Count*	*Average*	*Median*	*Standard deviation*	*Minimum*	*Maximum*	*Range*	*Low quartile*	*Upper quartile*	*Interquartile range*
herd 1	10	442.3	445.5	110.481	293.0	629.0	336.0	357.0	477.0	120.0
herd 2	10	501.5	492.0	173.123	230.0	849.0	619.0	378.0	567.0	189.0
herd 3	10	630.5	713.0	226.924	169.0	891.0	722.0	474.0	769.0	295.0
herd 4	10	491.8	481.0	98.1708	396.0	669.0	273.0	402.0	533.0	131.0
herd 5	10	705.6	520.0	522.303	423.0	2132.0	1709.0	453.0	605.0	152.0
Total	50	554.34	485.5	280.828	169.0	2132.0	1963.0	419.0	629.0	210.0

Chao richness estimation

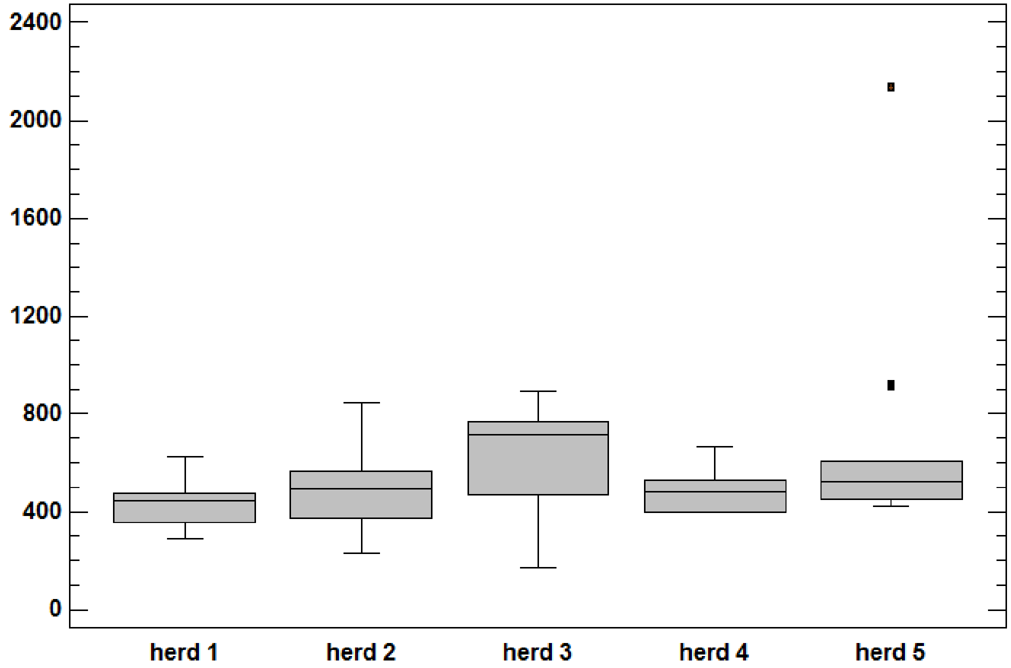

b) Chao Richness Estimation for pregnancy status

Pregnant status	*Count*	*Average*	*Median*	*Standard deviation*	*Minimum*	*Maximum*	*Range*	*Low quartile*	*Upper quartile*	*Interquartile range*
NON-PREGNANT	30	584.7	516.5	339.65	169.0	2132.0	1963.0	419.0	657.0	238.0
PREGNANT	20	508.8	481.0	153.851	293.0	919.0	626.0	415.0	594.0	179.0
Total	50	554.34	485.5	280.828	169.0	2132.0	1963.0	419.0	629.0	210.0

Chao richness estimation

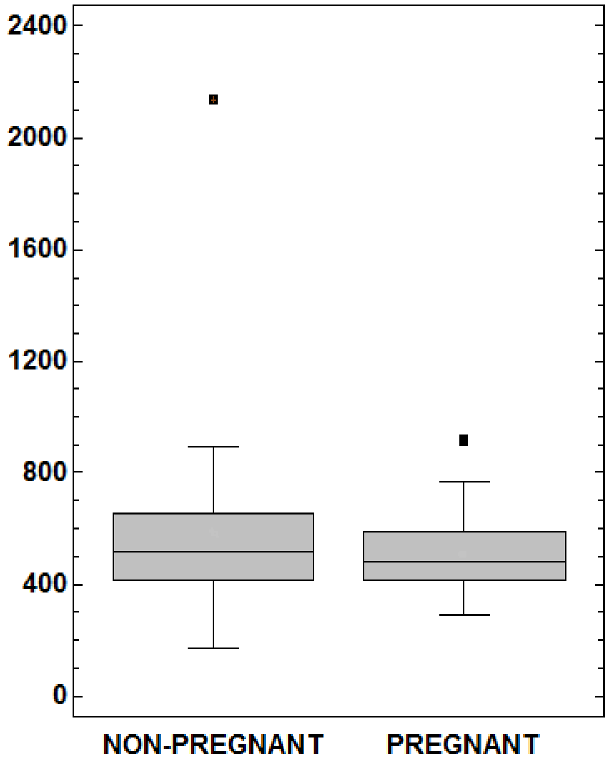

Supplement: Supplementary file 1 [file high-throughput-09-00016-s001.zip › Figure S3.pdf]
