# Supplementary figures and images for: Influence of the Ovine Genital Tract Microbiota on the Species Artificial Insemination Outcome. A Pilot Study in Commercial Sheep Farms
Source: High Throughput. 2020 Jul 6;9(3):16. doi: 10.3390/ht9030016 (PMC7576495; doi:10.3390/ht9030016)

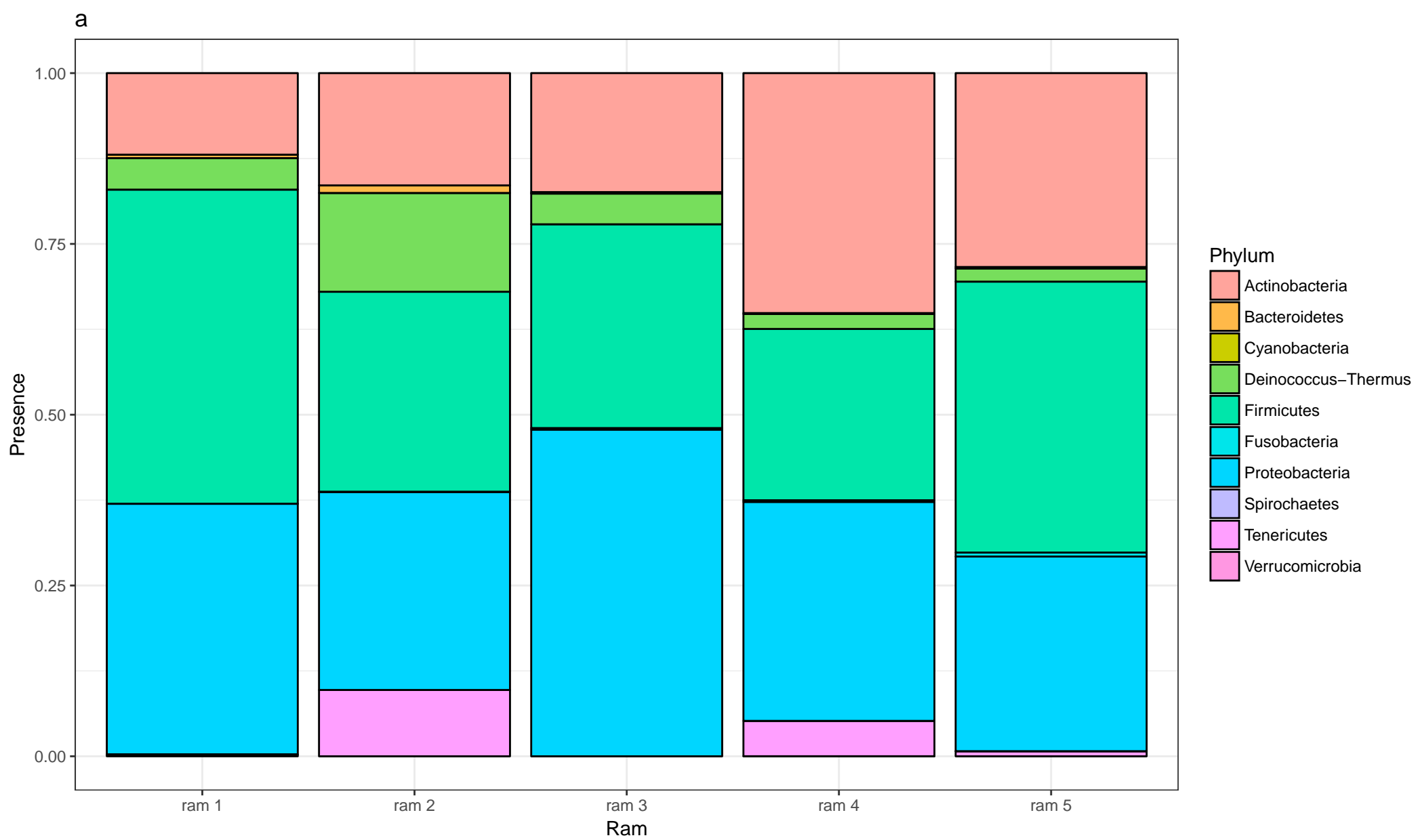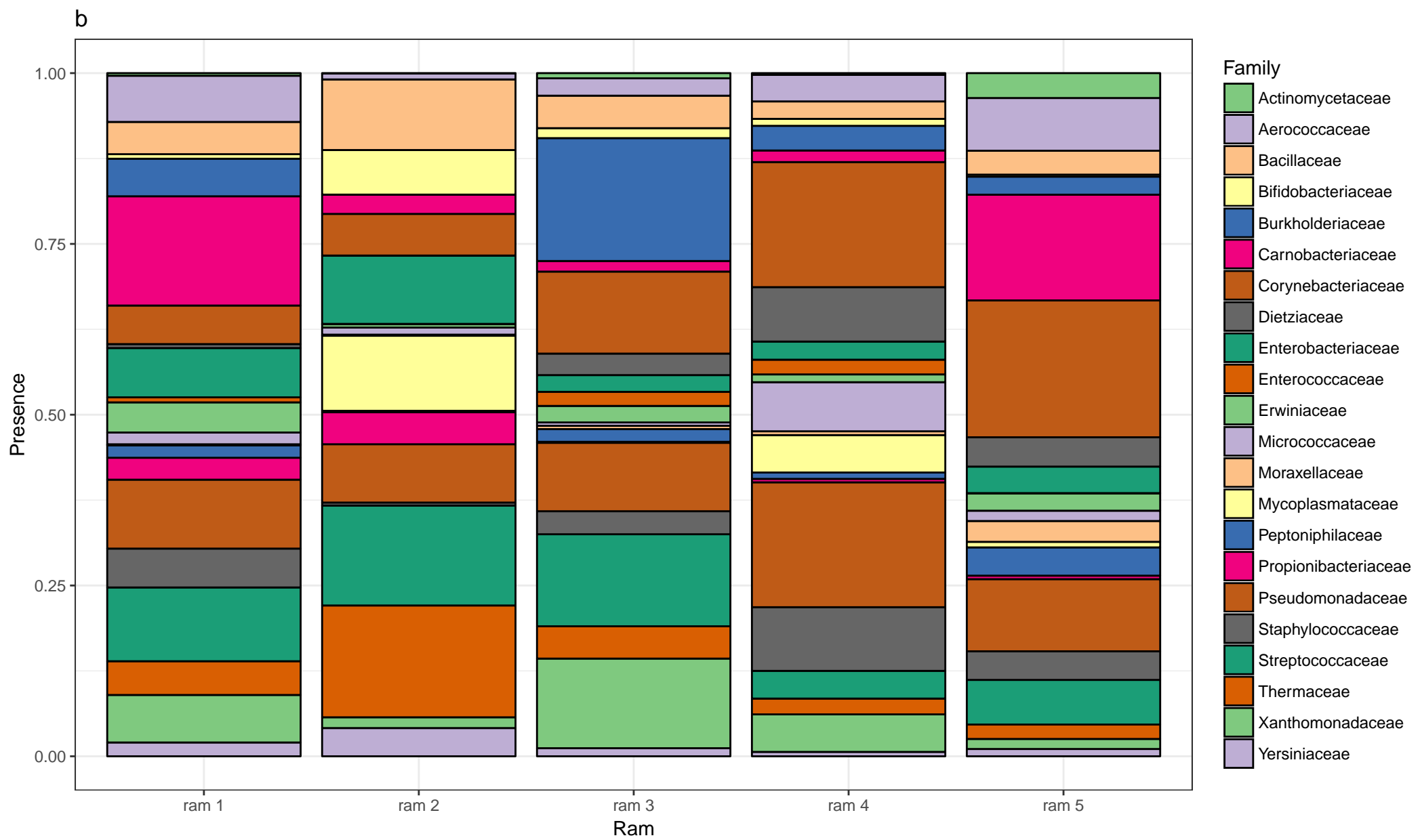

Supplement: Supplementary file 1 [file high-throughput-09-00016-s001.zip › Figure S4.pdf]

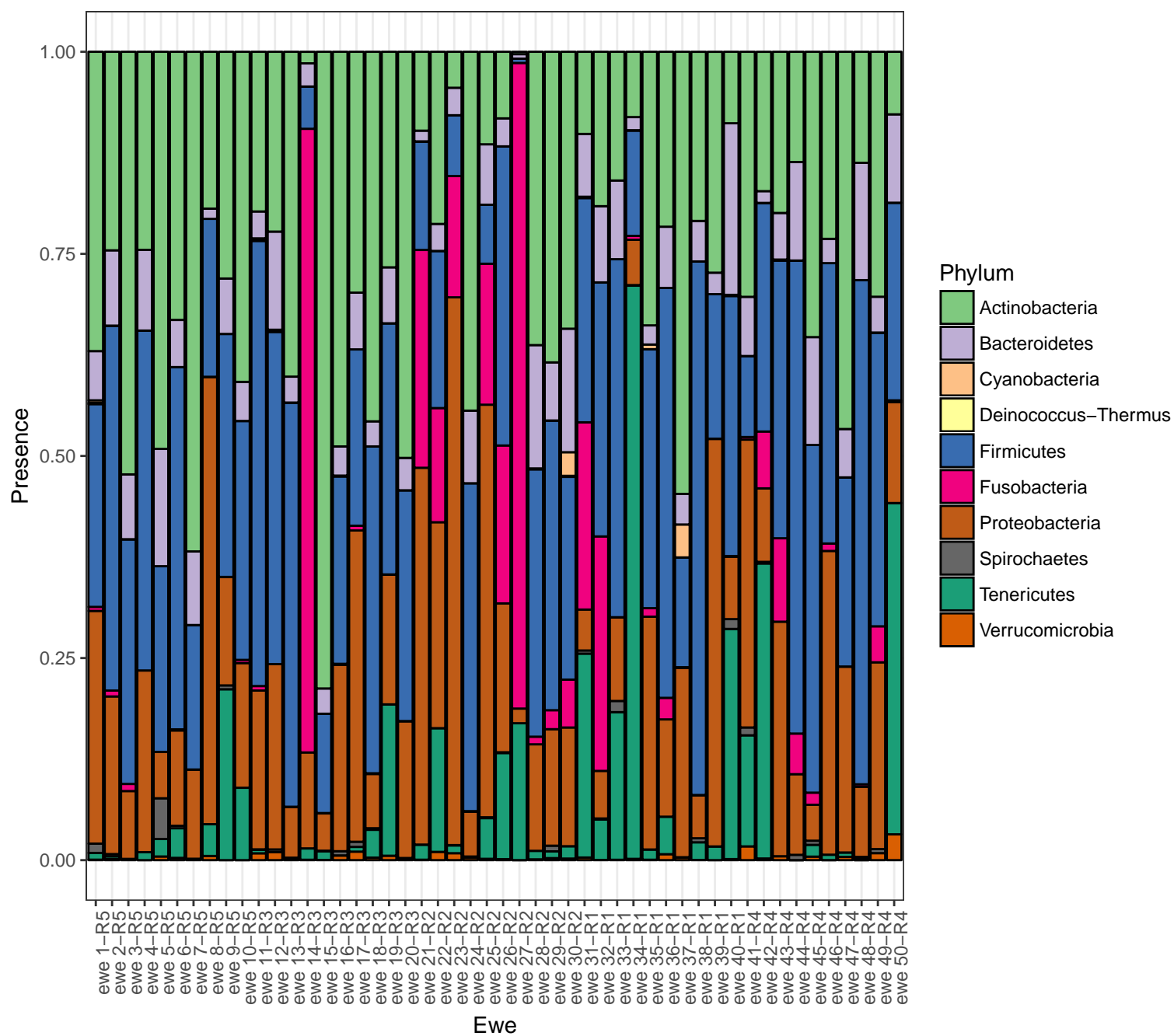

Supplement: Supplementary file 1 [file high-throughput-09-00016-s001.zip › Figure S5.pdf]

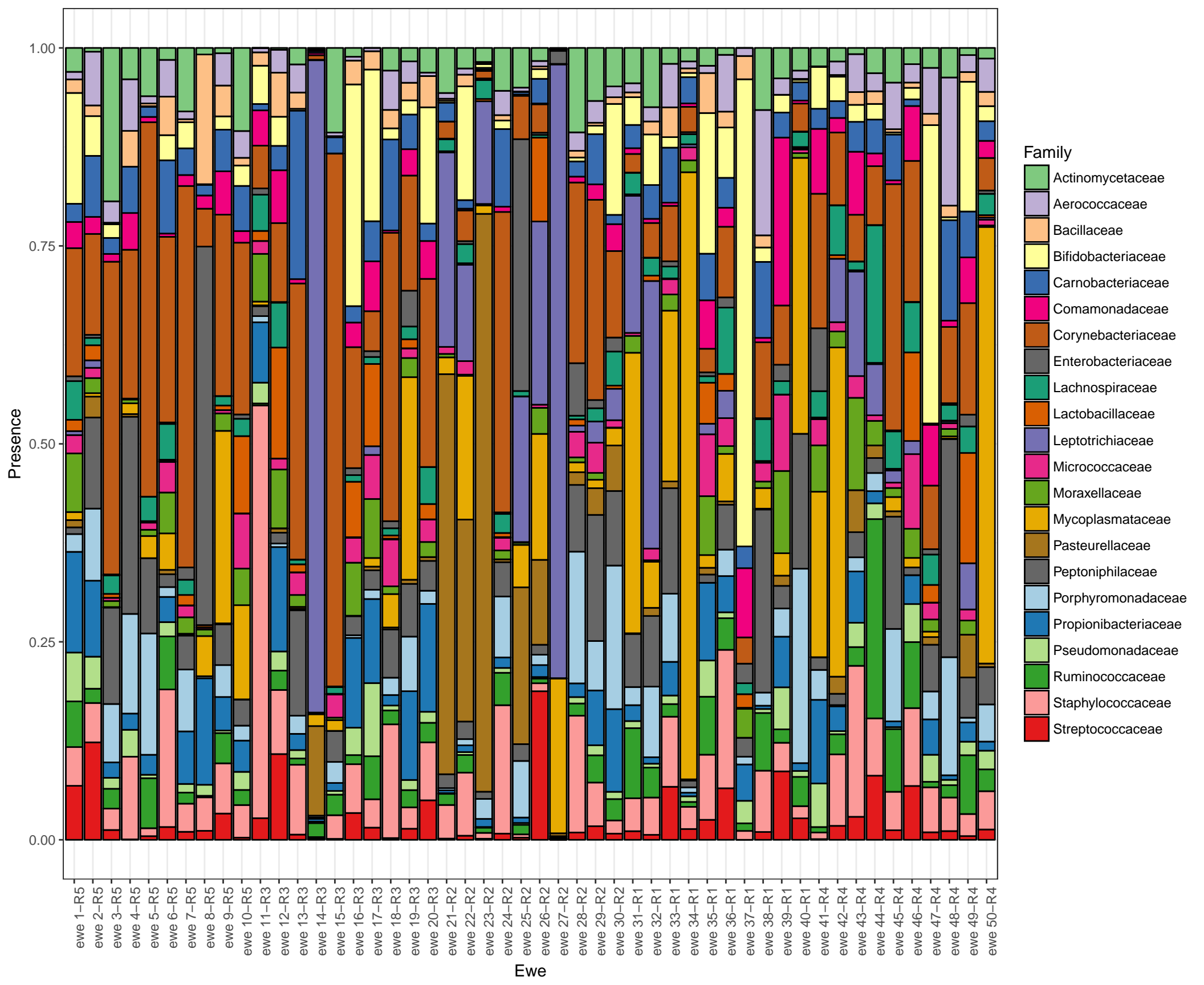

Supplement: Supplementary file 1 [file high-throughput-09-00016-s001.zip › Figure S6.pdf]

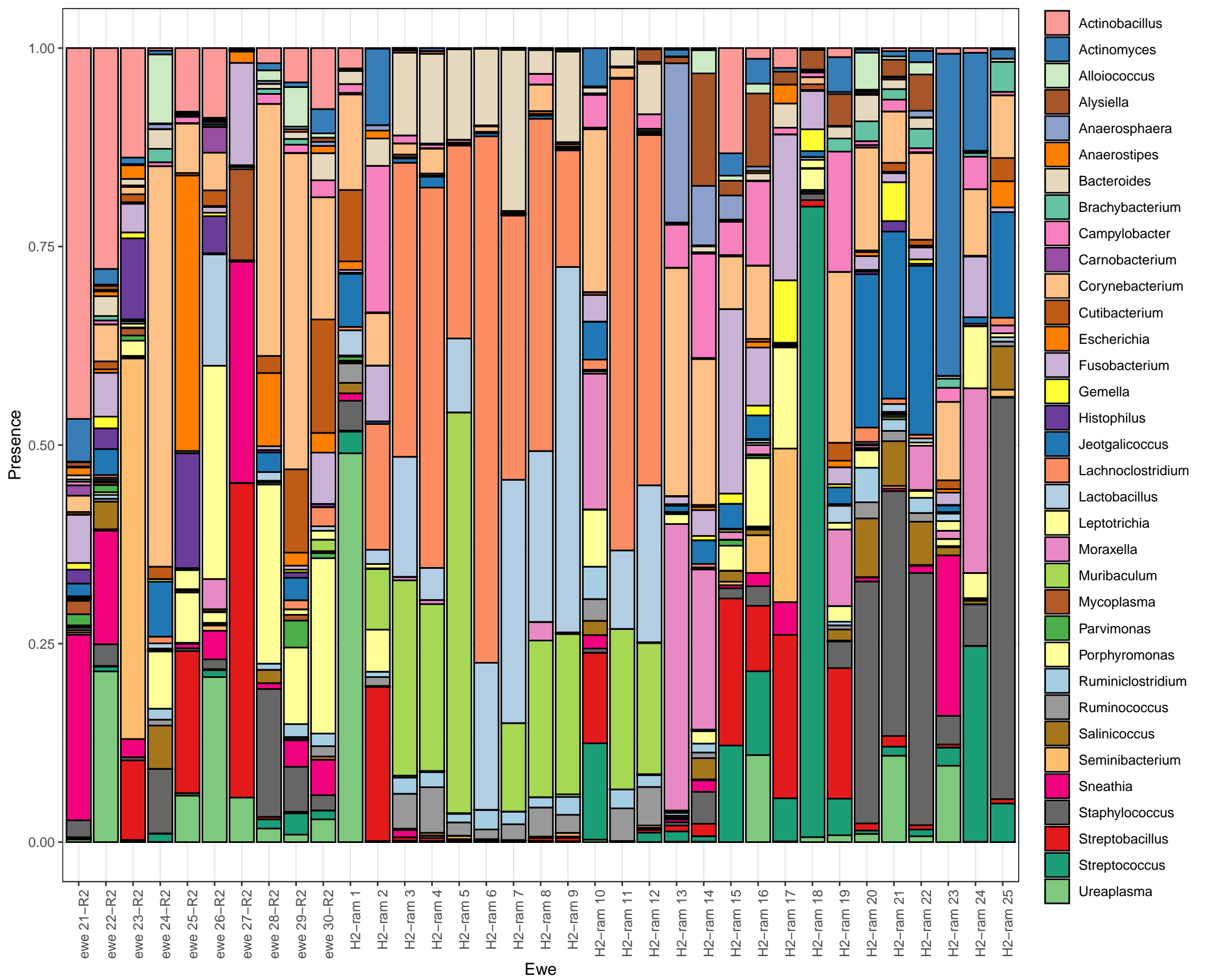

Supplement: Supplementary file 1 [file high-throughput-09-00016-s001.zip › Figure S7.pdf]
